# Use of Pharmacy Sales Data to Assess Changes in Prescription- and Payment-Related Factors that Promote Adherence to Medications Commonly Used to Treat Hypertension, 2009 and 2014

**DOI:** 10.1371/journal.pone.0159366

**Published:** 2016-07-18

**Authors:** Matthew Ritchey, Stavros Tsipas, Fleetwood Loustalot, Gregory Wozniak

**Affiliations:** 1 Division for Heart Disease and Stroke Prevention, National Center for Chronic Disease Prevention and Health Promotion, Centers for Disease Control and Prevention, Atlanta, Georgia, United States of America; 2 Health Outcomes Group, American Medical Association, Chicago, Illinois, United States of America; University of Perugia, ITALY

## Abstract

**Background:**

Effective hypertension management often necessitates patients’ adherence to the blood pressure (BP)-lowering medication regimen they are prescribed. Patients’ adherence to that regimen can be affected by prescription- and payment-related factors that are typically controlled by prescribers, filling pharmacies, pharmacy benefit managers, and/or patients’ health insurance plans. This study describes patterns and changes from 2009 to 2014 in factors that the literature reports are associated with increased adherence to BP-lowering medication.

**Methods and Findings:**

We use a robust source of United States prescription sales data—IMS Health’s National Prescription Audit—to describe BP-lowering medication fill counts and spending in 2009 compared with 2014. Moreover, we describe patterns and changes in adherence-promoting factors across age groups, payment sources, and medication classes. From 2009 to 2014, the BP-lowering medication prescription fill count increased from 613.7 million to 653.0 million. Encouraging changes in adherence-promoting factors included: the share of generic fills increased from 82.5% to 95.0%; average days’ supply per fill increased from 45.9 to 51.8 days; and average total (patient contribution) spending per years’ supply decreased from $359 ($54) to $311 ($37). Possibly undesirable changes included: the percentage of fills for fixed-dose combinations decreased from 17.1% to 14.2% and acquired via mail order decreased from 10.7% to 8.2%. In 2014: 653.0 million fills occurred accounting for $28.81B in spending; adults aged 45–64 years had the highest percentage of fixed-dose combinations fills (16.9%); and fills with Medicaid as the payment source had the lowest average patient spending per fill ($1.19).

**Conclusions:**

We identified both encouraging and possibly undesirable patterns and changes from 2009 to 2014 in factors that promote adherence to BP-lowering medications during this period. Continued tracking of these metrics using pharmacy sales data can help identify areas that can be addressed by clinical and policy interventions to improve adherence for medications commonly used to treat hypertension.

## Introduction

In 2011, the estimated cost of health care services, medications, and missed days of work associated with high blood pressure (BP) in the United States was $46 billion [1]. Despite this high cost and the wide availability of medication to lower BP [2–4], only three-fourths (53 million) of the estimated 70 million US adults with hypertension are being treated using BP-lowering medication and just over one-half (36 million) have their BP controlled [5]. The effective management of hypertension, resulting in a reduction in BP through the prescription and use of medication, in conjunction with diet and lifestyle modifications, has been shown to greatly decrease the incidence of and mortality from stroke, heart attack, and heart failure [6–9].

The World Health Organization states that poor patient adherence to long-term therapies to treat chronic conditions, including hypertension, is one of the leading causes of negative health outcomes and increased health care costs worldwide [10]. Likewise, in the United States, poor adherence to BP-lowering medication has been identified as a leading cause of ineffective BP control among hypertensive adults, as well as a significant risk factor for negative cardiovascular disease outcomes and increased health care costs [11–17]. The reasons for poor adherence to chronic disease medications, including medications that lower BP, in the United States are numerous and complex [18] and include several prescription- and payment-related factors that are associated with improved adherence. These factors, which are often controlled by prescribers, filling pharmacies, pharmacy benefit managers, and/or patients’ health insurance plans, include: reducing patient out-of-pocket burden with low copayments (copays) [19,20], increasing generic medication use [21], prescribing fixed-dose combination pills with more than one BP-lowering medication per pill [22–24], limiting the number of pills to take per day [25–27], increasing the days’ supply per fill—for example, 90-day vs 30-day allotments [25], and using mail order pharmacies [28,29]. However, we are unaware of any publications that have described national patterns or changes over time in these factors as they relate to BP-lowering medications.

This repeated cross-sectional study uses a timely and robust pharmacy sales data source of outpatient prescription medications filled in the United States to describe BP-lowering medication fill counts and spending in 2009 compared with 2014. Moreover, we describe how evidence-based prescription- and payment-related factors that promote adherence to these medications are represented across age groups, payment sources, and medication classes during this period. This information is expected to help inform the decisions of clinicians, health system administrators, payers, and policy makers related to these factors, potentially leading to population-level improvements in medication adherence and, in turn, improvements in BP control and cardiovascular disease outcomes, and lower health care costs.

## Materials and Methods

This study was determined by the Centers for Disease Control and Prevention Human Subjects Review Board to be for public health practice (surveillance) and exempt from IRB review. We obtained national annual outpatient prescription fill data from the IMS Health National Prescription Audit (NPA) for 2009 and 2014. The NPA contains information regarding outpatient prescription medication fills from retail and mail order pharmacies, and includes prescription information from ~87% of the retail universe and ~70% of the mail order universe in the United States [30]. IMS Health applies a patented projection methodology to the collected data to create the national level estimates within the NPA; these estimates do not include a sampling error. IMS Health data have previously been used to describe BP-lowering medication fills in other countries [31–33] and fills for other types of prescription medications in the United States [34–36].

The data consist of counts of total prescription fills, the number of days covered by the fills, the average quantity of pills dispensed per fill, and the average dollars spent per fill at the medication class level, with breakouts by distribution channel (retail and mail order), medication brand and generic status, patient age and sex, patient copay range, and prescription medication payment source, including commercial insurance plans (private insurance), one of the two main government-sponsored insurance plans, Medicaid or Medicare Part D, or out of pocket by the patient. We identified BP-lowering medication fills using IMS Health’s Uniform System of Classification schema and considered fills only among adults aged ≥18 years. We grouped the fills into the following medication class categories: angiotensin converting enzyme inhibitors (ACEIs); angiotensin II receptor blockers (ARBs); beta blockers (BBs); calcium channel blockers (CCBs); diuretics; and other BP-lowering medication. We allocated fills that contained more than one BP-lowering medication per pill (i.e., fixed-dosed combinations) into each appropriate medication class category.

We examined patterns and changes from 2009 to 2014 in several prescription-related factors that potentially promote adherence to BP-lowering medication by age group, payment source, and/or medication class. We calculated the average days’ supply per fill, which represents the average length of time before a prescription would need to be refilled, by dividing the total number of days of therapy covered by the fills by the total number of fills. To derive the average number of pills taken per day of therapy, i.e., the number of pills a person would need to take each day to get the prescribed daily dose of a specific medication, we divided the average number of pills per fill by the average days’ supply per fill for that specific medication. And lastly, we present the percentage of fills that were obtained via the mail order distribution channel, generic, paid for completely out of pocket, and fixed-dose combinations; and the percentage of fills with low or no patient copays, defined as $5.00 or less.

We also examined several payment-related factors that potentially promote adherence to BP-lowering medication. Total patient payment represents the spending patients incurred paying for fills completely out of pocket added to the estimated amount patients spent on copays. We estimated patient copay amounts to be the midpoint of each IMS Health copay category. For example, for the $.01 to $5.00 copay category, all copays were set equal to $2.50. Fill claims with unknown copay amounts (9.2% of fills in 2009, 2.7% of fills in 2014) were redistributed in proportion to fills among each payment source with known copay amounts. Average spending per years’ supply represents the average amount spent by the health care system and by patients for a year’s worth of BP-lowering medication. Finally, we calculated the average price per generic and brand fill. These calculations did not take into account differences in the average days’ supply per fill that may exist between generic and brand fills.

## Results

**Fig 1:**
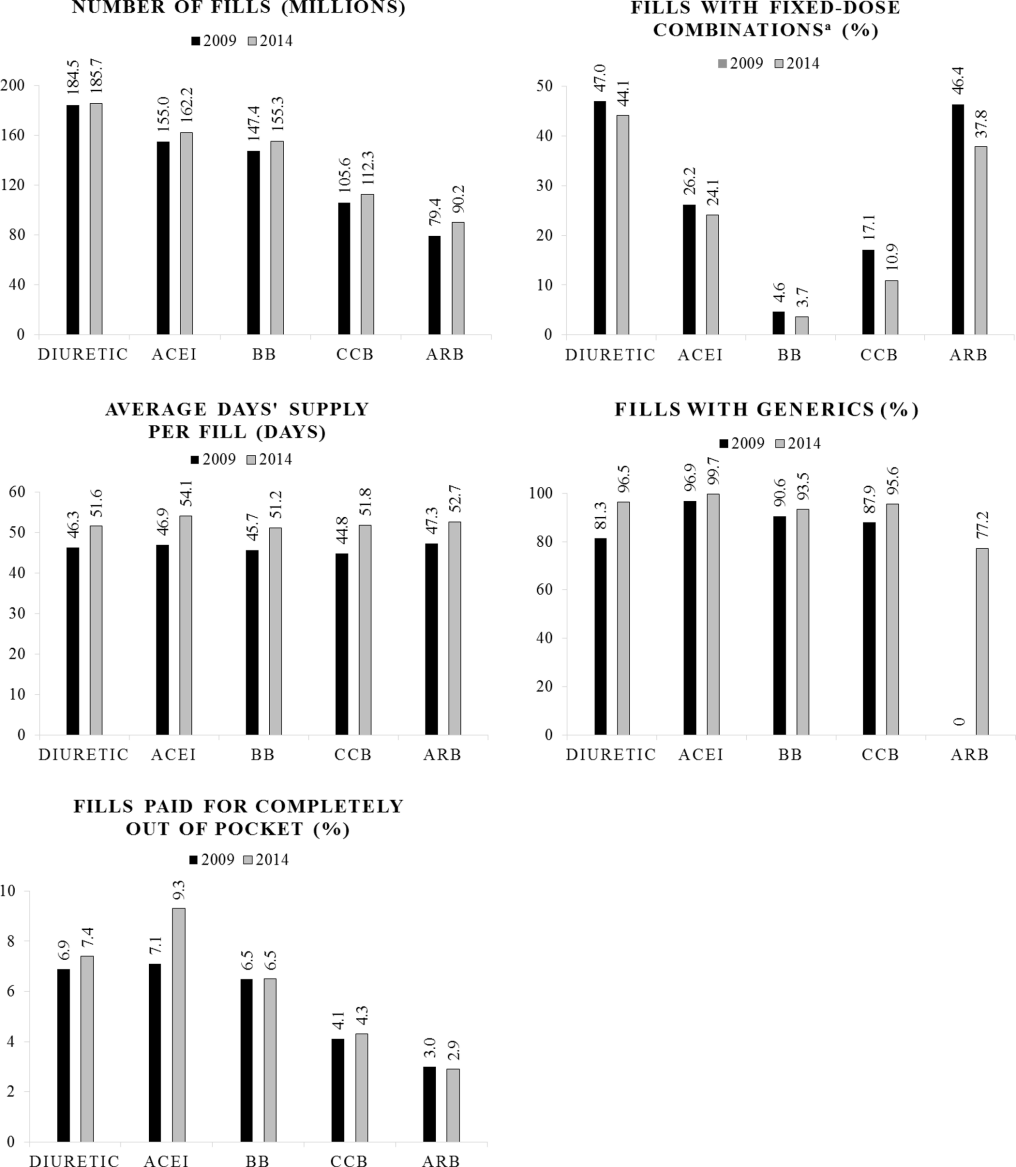
Prescription-related Factors that Promote Adherence to Blood Pressure-lowering Medications by Class, 2009 and 2014. ACEI indicates angiotensin converting enzyme inhibitor; BB, beta blocker; BP, blood pressure; CCB, calcium channel blocker; and, ARB, angiotensin II receptor blocker. ^a^ Fixed-dose combinations contain more than one active BP-lowering medication ingredient per pill. Source: IMS Health National Prescription Audit, 2009–2014.

From 2009 to 2014, BP-lowering medication prescription fill counts increased from 613.7 million to 653.0 million (Table 1). Total system spending on fills increased from $27.72B, in 2009, to $28.81B being spent in 2014. Of the 653.0 million fills in 2014, the most fills occurred among those aged 45–64 years (284.0 million) (Table 1). The percentage of fills that was for fixed-dose combinations decreased from 17.1% in 2009 to 14.2% in 2014. In 2014, the percentage of fills for fixed-dosed combinations was highest among those aged 45–64 years (16.9%) (Table 1), patients who paid for the fill completely out of pocket (16.6%) (Table 2), or where the fill contained a diuretic (44.1%) (Fig 1). The average number of pills taken per day of therapy remained relatively consistent over time and across age groups (Table 1). In 2014, among adults aged ≥18 years, an average of 1.11 pills per day was needed to achieve a daily therapeutic dose.

**Table 1:** Prescription- and payment-related Factors that Promote Adherence to Blood Pressure-lowering Medications by Age Group, 2009 and 2014.

** **	** **	**Age Group (y)**					
**Measure**	**Year**	**18–44**	**45–64**	**65–74**	**≥75**	**Unknown**	**Total**
**Total blood pressure-lowering medication fills (millions)**							
	2009	53.7	254.5	136.6	151.9	17.1	613.7
	2014	62.8	284.0	152.7	143.6	9.9	653.0
**Fills with fixed-dose combinations**^**a**^ **(%)**							
	2009	18.9	20.4	16.0	12.0	15.5	17.1
	2014	14.8	16.9	13.6	9.5	12.0	14.2
**Average number of pills taken per day of therapy**^**b**^							
	2009	1.16	1.09	1.09	1.11	1.09	1.11
	2014	1.15	1.10	1.10	1.13	1.10	1.11
**Average days' supply per fill**^**c**^ **by outlet (days)**							
All fills	2009	37.2	44.5	49.9	48.7	43.8	45.9
	2014	41.1	48.1	57.9	57.8	48.1	51.8
Retail outlet fills	2009	34.8	38.4	43.2	42.5	43.8	40.1
	2014	39.6	45.0	55.0	54.7	48.1	48.9
Mail order outlet fills	2009	83.4	85.5	87.1	87.1	86.3	86.3
	2014	82.7	84.1	85.7	85.9	63.6	85.0
**Fills acquired via mail order (%)**							
	2009	4.6	10.9	13.0	11.8	0.0	10.7
	2014	3.3	7.8	9.7	9.9	0.0	8.2
**Fills paid for completely out of pocket (%)**							
	2009	8.4	6.4	4.5	4.5	17.8	6.0
	2014	10.0	7.9	4.8	3.9	2.6	6.4

^a^ Fixed-dose combinations contain more than one active blood pressure-lowering medication ingredient per pill.

^b^ Represents the number of pills a person would need to take each day to get the prescribed daily therapeutic dose.

^c^ Represents the average length of time before a prescription would need to be refilled.

Source: IMS Health National Prescription Audit, 2009–2014.

**Table 2:** Prescription- and payment-related Factors that Promote Adherence to Blood Pressure-lowering Medications by Payment Source, 2009 and 2014.

		Payment Source^a^				
	**Year**	**Out of Pocket by Patient**	**Commercial Plan**	**Medicaid**	**Medicare Part D**	**Total**
Number of blood pressure-lowering medication fills (millions)						
	2009	37.0	388.2	22.6	165.9	613.7
	2014	42.0	379.9	14.3	216.8	653.0
Fills with fixed-dose combinations^b^ (%)						
	2009	16.4	19.1	13.0	13.0	17.1
	2014	16.6	16.1	10.3	10.7	14.2
Fills that were for generic medications (%)						
	2009	90.3	80.2	85.3	86.0	82.5
	2014	98.1	94.2	96.0	95.8	95.0
Fills with a low or no copay^c^ (%)						
	2009	—	68.4	99.0	82.7	73.8
	2014	—	76.5	99.5	84.8	79.9
Estimated average patient spending per fill ($US)						
	2009	29.94	5.87	1.00	4.23	4.89
	2014	24.73	4.13	1.19	3.56	5.20

^a^ Includes fills paid for entirely out of pocket by the patient or billed to a commercial insurance plan (private insurance) or one of the two main government-sponsored insurance plans, Medicaid or Medicare Part D.

^b^ Fixed-dose combinations contain more than one active blood pressure-lowering medication ingredient per pill.

^c^ Copay of $5.00 or less.

Source: IMS Health National Prescription Audit, 2009–2014.

The average days’ supply per fill for all fills increased from 45.9 days in 2009 to 51.8 days in 2014 (Table 1). While the average days’ supply per fill remained considerably higher in mail order compared with retail fills, the overall increase in average days’ supply per fill was primarily driven by increases in the average days’ supply dispensed via the retail distribution channel (Table 1). The days’ supply per fill for all fills increased across each age category and, in 2014, was highest among those aged 65–74 years (57.9 days) and lowest among those aged 18–44 years (41.1 days). These values increased for every medication class from 2009 to 2014 (Fig 1). In 2014, among the medication classes, fills for ACEIs had the highest average days’ supply per fill (54.1). The percentage of fills acquired by mail order decreased slightly from 10.7% in 2009 to 8.2% in 2014 (Table 1). In 2014, adults aged 18–44 years used mail order the least frequently (3.3%), and adults aged ≥ 75 used mail order the most frequently (9.9%).

Overall, the proportion of fills representing generic BP-lowering medication increased from 82.5% in 2009 to 95.0% in 2014 (Table 2). Generics were used for 85.3% to 96.0% of Medicaid fills throughout the period. In contrast, the lowest proportion of fills for generics was in commercial insurance plans (80.2% in 2009 and 94.2% in 2014). The percentage of fills for generics increased for every medication class over time (Fig 1). Most notably, the percentage of fills for ARB generics increased from 0% in 2009 to 77.2% in 2014. This corresponded with an almost 14% increase in the total number of fills for ARBs, from 79.4 million, in 2009, to 90.2 million, in 2014; representing the greatest percent increase from 2009 to 2014 in fills seen within the observed medication classes.

Comparing 2009 to 2014, the percentage of fills paid for completely out of pocket increased across all age groups except for those aged ≥75 years, where it decreased from 4.5% to 3.9% (Table 1). Patients aged 18–44 years had the highest percentage of fills paid for completely out of pocket in 2009 and in 2014, 8.4% and 10.0%, respectively. There was a slight variation by medication class in the percentage of fills that were paid for completely out of pocket by patients (Fig 1). In 2014, this percentage was highest for ACEIs (9.3%) and diuretics (7.4%) and lowest for ARBs (2.9%).

About 99% of Medicaid fills had low or no patient copays throughout the period, while only 68.4% in 2009 and 76.5% in 2014 of commercial insurance plan fills had low or no copays (Table 2). Estimated average patient spending per fill was lowest for Medicaid fills (approximately $1.00) over the period. Fills paid for by commercial plans had the highest average copays: $5.87 in 2009 and $4.13 in 2014. Patients spent an average of $24.73 per fill in 2014 on prescriptions they paid for completely out of pocket compared to $29.94 in 2009.

**Fig 2:**
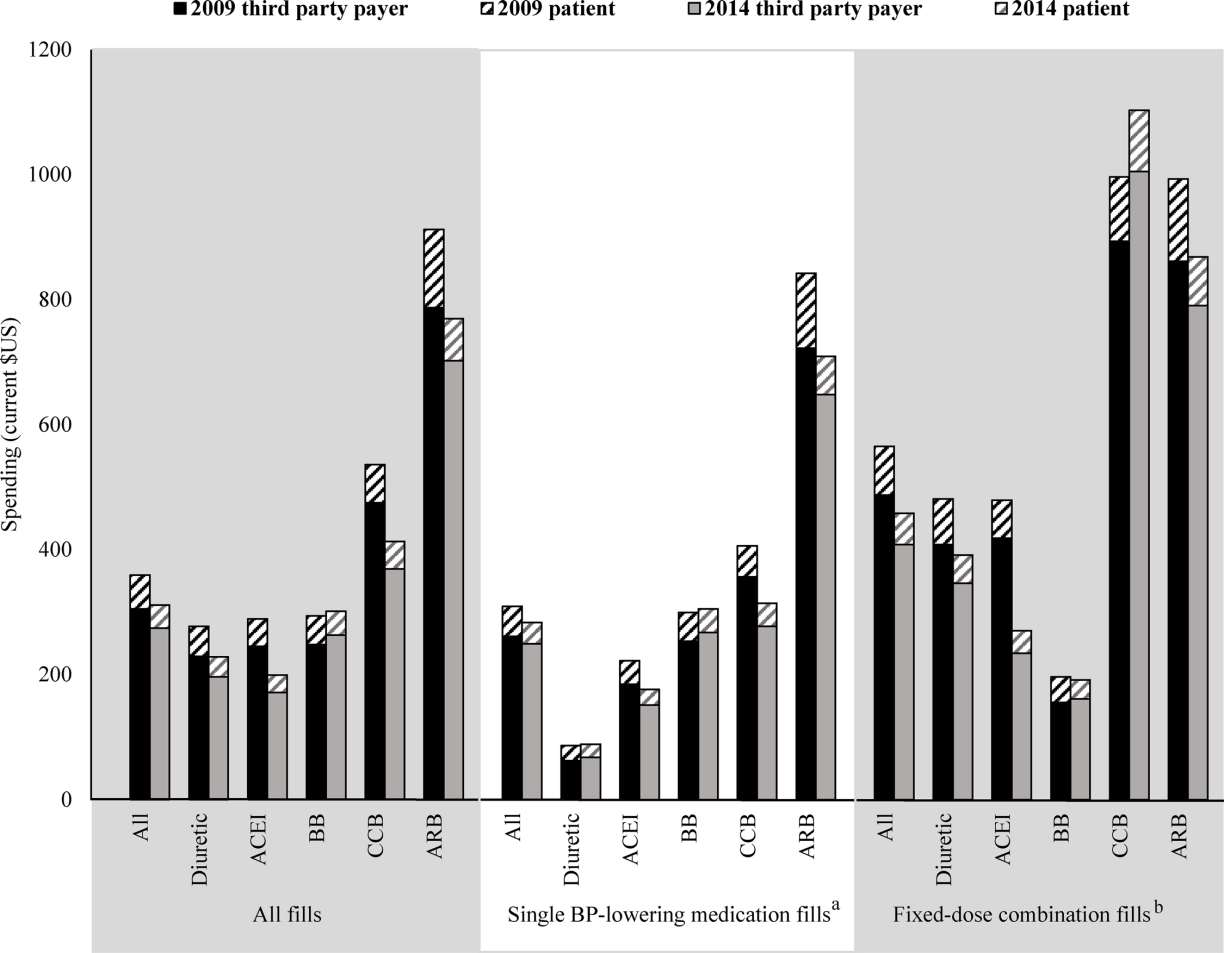
Payment-related Factors that Promote Adherence to Blood Pressure-lowering Medications by Class, 2009 and 2014. ACEI indicates angiotensin converting enzyme inhibitor; BB, beta blocker; BP, blood pressure; CCB, calcium channel blocker; and, ARB, angiotensin II receptor blocker. ^a^ Single medication fills contain one active BP-lowering medication ingredient per pill. ^b^ Fixed-dose combinations contain more than one active BP-lowering medication ingredient per pill. Source: IMS Health National Prescription Audit, 2009–2014.

**Fig 3:**
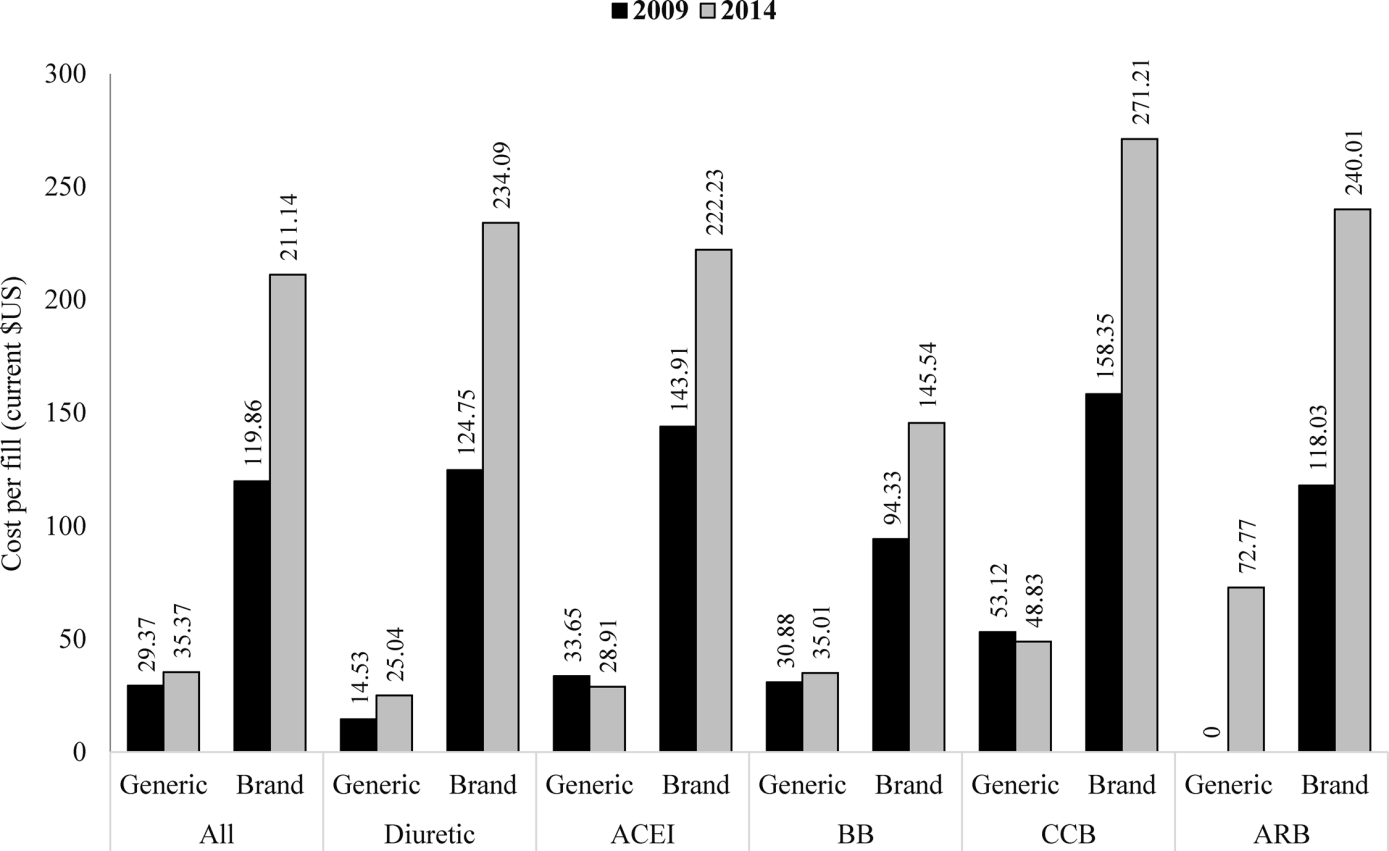
Cost Per Fill of Generic versus Brand Prescriptions for Medications Commonly Used to Lower Blood Pressure, 2009 and 2014. ACEI indicates angiotensin converting enzyme inhibitor; BB, beta blocker; BP, blood pressure; CCB, calcium channel blocker; and, ARB, angiotensin II receptor blocker. Source: IMS Health National Prescription Audit, 2009–2014.

In 2014, fills containing ARBs had the highest average price per years’ supply ($769; $912 in 2009) (Fig 2). This was almost four times the average price per years’ supply for ACEI fills ($199; $289 in 2009). To estimate the average price per years’ supply for each individual BP-lowering medication type, the prices for fixed-dose combination and single medication pill fills were assessed separately. Again, in 2014, ARB-only fills (excludes fixed-dosed combination ARB fills) had the highest average price per years’ supply ($709; $842 in 2009), with the patient being responsible for $61 of that total per year ($120 in 2009). Diuretic-only fills had the lowest average price per years’ supply in 2014 ($88), with the patient being responsible for $21 of that total per year. Unlike the other medication classes that experienced considerable decreases in per years’ supply spending for single medication pills from 2009 to 2014, spending on diuretic-only and beta blocker-only fills showed little change. Diuretic-only fills had the lowest average price per years’ supply in 2009 ($86) and in 2014 ($88). Patients were responsible for $24 and $21 of the total per year, respectively. Beta blocker-only fills had an average price per years’ supply of $299 in 2009 and $305 in 2014. Patients’ responsibility was $46 and $38 of that total per year, respectively. There was a considerable difference in the price per fill for generic versus brand medications (Fig 3). From 2009 to 2014, the brand-to-generic price ratio increased from 4.1 ($119.86/$29.37) to 6.0 ($211.14/$35.37) for all BP-lowering medications. Among the mediation classes, ARBs had the highest average generic price per fill in 2014 ($72.77) and the CCBs had the highest average brand price per fill ($271.21).

## Discussion

The literature has demonstrated that patients’ adherence to their prescribed chronic disease medication regimens can be significantly impacted by the prescription- and payment-related factors assessed in this study [19–29]. However, prior to this study, we are unaware of any studies that have attempted to better understand and track these factors to help inform interventions that may positively address adherence to BP-lowering medications in the United States. Despite their wide availability—over 650 million prescriptions for BP-lowering medications were filled in 2014 alone—about half of U.S. adults with hypertension still do not have their BP under control [5]. This includes around 20 million US adults who, despite having a usual source of care (e.g., primary care physician), have uncontrolled blood pressure potentially due to poor medication adherence. This includes people who are aware of having hypertension, but are not currently taking medication to treat it, and people who are receiving treatment for hypertension, but the medication regimen or how they are adhering to it is insufficient to control their blood pressure [37]. Patients’ adherence to their BP-lowering medication regimens, including taking medications as directed and being persistent in their long-term use, is a central component of improving hypertension control and decreasing the burden of cardiovascular events in the United States [17].

Our use of IMS Health’s NPA pharmacy sales data to describe patterns and changes over time in factors that potentially promote adherence to BP-lowering medication revealed several encouraging findings. First, in 2014 compared with 2009, there were lower patient copays and an increase in the proportion of fills that were for generics across all BP-lowering medication classes. By 2014, the percentage of total fills with low or no copay increased to 79.9% and the percentage of fills that where for generic medications (which were approximately one-sixth the price of brand fills) increased to 95%. These factors may at least partially explain the 22% decrease in average patient spending from 2009 to 2014. Reducing patient out-of-pocket costs by lowering copays [20] and increasing generic medication use [21] are effective ways to improve medication adherence and are strategies suggested by the United States Community Preventive Services Task Force [19]. In addition, the average spending per years’ supply for most BP-lowering medications was low (average of $311 for all BP-lowering medications in 2014), especially for patients (average of $37 for all BP-lowering medication in 2014), and the average patient spending per years’ supply decreased in 2014 compared to 2009 for all medication classes assessed. However, for patients needing to take more than one medication to control their hypertension [4,12], average annual per-patient spending on medications to treat hypertension is likely higher. Finally, we observed a trend of increasing average days’ supply per fill across all classes. By moving to longer fill periods (e.g., 90 days’ supply fills) the frequency and complexity of filling medications are reduced for patients and adherence is expected to improve [25].

We also observed several possibly undesired changes. For example, the percentage of fills obtained via mail order pharmacies decreased from 10.7% to 8.2% between 2009 and 2014. However, while mail order pharmacy use to obtain medications has been shown to improve adherence to chronic disease-related medications [28,29], this relationship might be due to mail order fills typically having a greater average days’ supply than prescriptions obtained in retail outlets. This study found that the average days’ supply per fill remained high for prescriptions obtained via mail order, but increased considerably for medications obtained via the retail distribution channel during this period. This may be partially a result of large retail pharmacy chains offering low-price (e.g., $9.99–$14.99) 90-day generic fill options [38]. Therefore, the distribution channel type may be less important to adherence than is the number of days’ supply per fill. In addition, increased use of retail pharmacies creates the potential for additional opportunities for pharmacists’ to engage patients in face-to-face counseling to help better manage their medication regimens, a strategy that has been associated with improved adherence [39]. Therefore, the reduction in mail order use, at least in some settings, may actually be an encouraging finding. The movement towards pharmacy chains offering low-priced generics that are purchased directly by patients, without third-party payer involvement, also appeared to be one of the factors that led to an increase in the percentage of fills paid for completely out of pocket by patients. However, despite this increase the overall average patient spending per fill for fills paid for completely out of pocket decreased by over $5 from 2009 to 2014. Therefore, this change likely did not negatively affect adherence, but may have promoted improved adherence.

Another possibly undesired trend was the 17% decrease in prescribing fixed-dose combination pills from 2009 to 2014. The percentage of adults who need to take multiple medications to control their hypertension has been increasing [4]. Prescribing fixed-dose combinations for patients who need to take multiple medications to control their hypertension is generally recommended because it reduces both the number of pills and the complexity of patient regimens, thereby improving adherence [22–24]. This is likely even more important among older patients and those covered by public health insurance, who often need to manage multiple chronic conditions [40,41] and might take more than one medication to manage their hypertension [4]. However, in 2014, among adults aged ≥75 years, less than 10% of the fills were for fixed-dose combinations. Moreover, among adults with government-sponsored prescription medication coverage (i.e., Medicaid or Medicare Part D) as their primary payment source, only 10.3% and 10.7%, respectively, of the fills were for fixed-dose combinations. Finally, limiting the average number of pills taken each day has been another strategy suggested to reduce therapeutic complexity and improve adherence [25–27]. While the number of pills needed to achieve a daily therapeutic dose remained low in this study (1.11 pills each day in 2014), there was no improvement noted from 2009 to 2014.

There were potential limitations to this study. First, we were unable to assess the appropriateness of or intended reason for prescribing, because our data did not capture health care visits and diagnoses. Therefore, BP-lowering medication fill counts and spending cannot be entirely attributed to the treatment of hypertension, and trends in the adherence-promoting factors may differ depending on the intended use of the medication. Second, fill data may not represent actual use, because patient adherence to treatment regimens varies. Third, because the patient copay information was collected by category and not as dollar amounts, we had to estimate patient-level spending by using the midpoint value of each copay category. Moreover, we estimated fills with unknown copay amounts in proportion to fills where that information was known, possibly redistributing fills to incorrect categories. However, by 2014, the amount of this type of missing data was minimal (<3% of fills). Fourth, there may be been misclassification of the payment source for some fills. For example, fills paid for using Medicare Advantage Part D plans may have been classified as having a commercial payment source, and we may have underestimated fills paid for by Medicare Part D. Fifth, we likely underestimate the patients’ average spending per years’ supply as our data only captures copay-related spending and no other patient spending, including meeting deductibles. Finally, because these data represent fills obtained via retail and mail order distribution channels, they may be missing BP-lowering medication fill data relating to patients seen within systems that have their own outpatient pharmacies, such as the Department of Veterans’ Affairs, integrated private sector delivery systems, and some safety net systems. Therefore, our overall fill estimates may be conservative.

Our study demonstrates that the use of pharmacy sales data (e.g., NPA) can be valuable to the public health and healthcare sectors in allowing for the timely assessment of prescription- and payment-related promoters of adherence to BP-lowering medications. Most analyses of medication prescribing and use rely on health insurance plan administrative data, which can have a considerable lag in reporting time and omits fills paid for completely out of pocket by patients. In comparison, the NPA data presented here are available with a 3-month time lag and represents projected fill counts across the retail and mail order distribution channels. These counts capture information on fills for low-price generic prescriptions that are paid for by patients completely out of pocket and that may be used more frequently as employers increasingly offer only high-deductible health plans [42]. Additional analyses of pharmacy sales data could look at similar trends in adherence promoting factors for other chronic disease medications. In addition, future studies should explore how changes in the factors described within this paper, both individually and in combination, are associated with population-level improvements in BP-lowering medication adherence, hypertension control, and cardiovascular disease outcomes. For example, despite the considerable improvement in hypertension control rates that occurred among US adults from 1999 (31.5%) to 2009 (53.3%), minimal improvement occurred during our study period from 2009 to 2014 (54.0%) [43]. Therefore, the mixture of the desired and undesired changes in the adherence promotion factors reported on in this study, in combination with other factors not assessed here, appeared to have only led to a slight improvement in blood pressure control during this period.

Considerably more work needs to be done to meet the Healthy People 2020 goal of 61.2% of hypertensive US adults having their blood pressure controlled [44]. The United States Department of Health and Human Services’ Million Hearts [45] initiative and the American Medical Association’s Improving Health Outcomes [46] initiative aim to improve hypertension control by promoting use of evidence-based strategies and standardized hypertension treatment approaches in clinical practice [47]. Implementing such approaches across health care delivery systems, payers, and practices should reduce variability in prescribing habits based on nonclinical factors, simplify treatment regimens, and encourage improvements being made in the adherence promotion factors discussed in the study, thus leading to more efficient and cost-effective therapy [22,47,48].

The ultimate public health goal is to decrease the prevalence of hypertension over time through population-level improvements in diet- and lifestyle-related risk factors, including decreasing dietary sodium intake, increasing physical activity levels, and increasing the prevalence of people with a healthy weight status [45]. However, clinicians, health care system administrators, payers, pharmacy benefit managers, and policy makers should also work together to establish mechanisms and policies to ensure that people with hypertension are diagnosed, be made aware of their diagnosis, and have their elevated BP appropriately treated. One objective of these efforts would be to improve factors that enhance patients’ ability to remain adherent to their medication regimens, including ones related to the prescription- and payment-related factors described within this study. Collective improvements in these factors for BP-lowering medication should track improved adherence and be accompanied by better BP control, improved short- and long-term cardiovascular outcomes, and reduced health care costs.
